# SARS-CoV-2 and the Angiotensin-Converting Enzyme 2 Receptor: Angiotensin-Converting Enzyme Inhibitor/Angiotensin 2 Receptor Blocker Utilization and a Shift Towards the Renin-Angiotensin-Aldosterone System Classical Pathway

**DOI:** 10.7759/cureus.55563

**Published:** 2024-03-05

**Authors:** Randy Felber, William New, Suzanne I Riskin

**Affiliations:** 1 Department of Foundational Sciences, Nova Southeastern University Dr. Kiran C. Patel College of Osteopathic Medicine, Clearwater, USA

**Keywords:** ace2 receptor, cardiovascular disease, antihypertensive, angiotensin receptor blockers, ace inhibitors, covid-19, coronavirus, renin-angiotensin-aldosterone system

## Abstract

Severe acute respiratory syndrome coronavirus 2 (SARS-CoV-2), the virus responsible for the coronavirus disease 2019 (COVID-19) pandemic, uses the surface angiotensin-converting enzyme 2 (ACE2) receptor as the site of entry into host cardiac, respiratory, intestinal, renal, and nervous system cells. Predisposing risk factors such as cardiovascular disease increase the risk of developing severe disease. Hypertension is characterized by the stimulation of the renin-angiotensin-aldosterone system (RAAS). Angiotensin-converting enzyme inhibitors (ACEis) and angiotensin 2 receptor blockers (ARBs), medications used to treat hypertension, inhibit RAAS and its downstream effects; however, they have also been shown to upregulate ACE2 receptors. In this review, we aim to evaluate the effectiveness of ACEi/ARBs as an adjunct therapy in patients with SARS-CoV-2 as well as examine the possible protective effects and impact on infection rate and disease severity.

A PubMed literature search excluding sources outside the United States and duplicates was performed using the following search criteria: “COVID-19 AND cardiovascular disease AND ACEi AND ARB”, “SARS-COVID-19 OR COVID-19, AND ACEi AND ARB AND Infection rate”, “COVID-19 AND ACEi and ARB”, “Omicron BA.1 and BA.2 AND ACE2 OR ARBs”, “Omicron AND ACEi AND ARBs”. This resulted in 33 final sources. The review concluded that ACEi/ARB therapy may continue to improve COVID-19 survival as previous treatment is associated with positive clinical outcomes. Patients taking ACEis or ARBs were found to have a decreased risk of hospitalization, reduced severity of COVID-19 pneumonia, a lesser need for mechanical ventilation, and an overall reduction in mortality rate. No statistically significant association between ACEi/ARB use and enhanced COVID-19 infectivity was found. The Omicron variant is theoretically more infectious and was associated with increased negative clinical outcomes in those undertreated with ACEis/ARBs. The majority of the literature supports the current guidelines from the American College of Cardiology (ACC), American Heart Association (AHA), European Society of Cardiology (ESC), and Heart Failure Society of America (HFSA), which state that ACEi and ARB medications should not be withdrawn from or initiated on patients with cardiovascular disease who are infected with SARS-CoV-2. More research needs to be conducted on the association between the emerging COVID-19 variants and ACEis/ARBs to give clinicians confidence when treating patients within this subgroup of the population.

## Introduction and background

The coronavirus disease 2019 (COVID-19) pandemic caused by severe acute respiratory syndrome coronavirus 2 (SARS-CoV-2) began in December 2019 in Wuhan, the capital city of Hubei Province in central China. The outbreak continues to impact global health and endanger the lives of millions across the world. Over time, the virus changed and formed different variants, the latest being Omicron, also known as XBB1.5, which rose to over 50% of the circulating lineages in December 2021 [1]. Predisposing risk factors such as advanced age, diabetes, and cardiovascular disease increase the risk of developing severe disease. The virus uses the surface-bound angiotensin-converting enzyme 2 (ACE2) as the site of entry into the host cells. The ACE2 is expressed on a variety of different cells, including those lining the respiratory tract, cardiac fibroblasts, cardiomyocytes, and vascular smooth muscle cells, as well as cells in the kidney, intestine, and central nervous system. The virus binds and fuses with the ACE2 receptor using its membrane-bound spike protein, taking the receptor into the cell with it. This fusion ultimately results in the downregulation of the ACE2 receptor [2].

**Figure 1:**
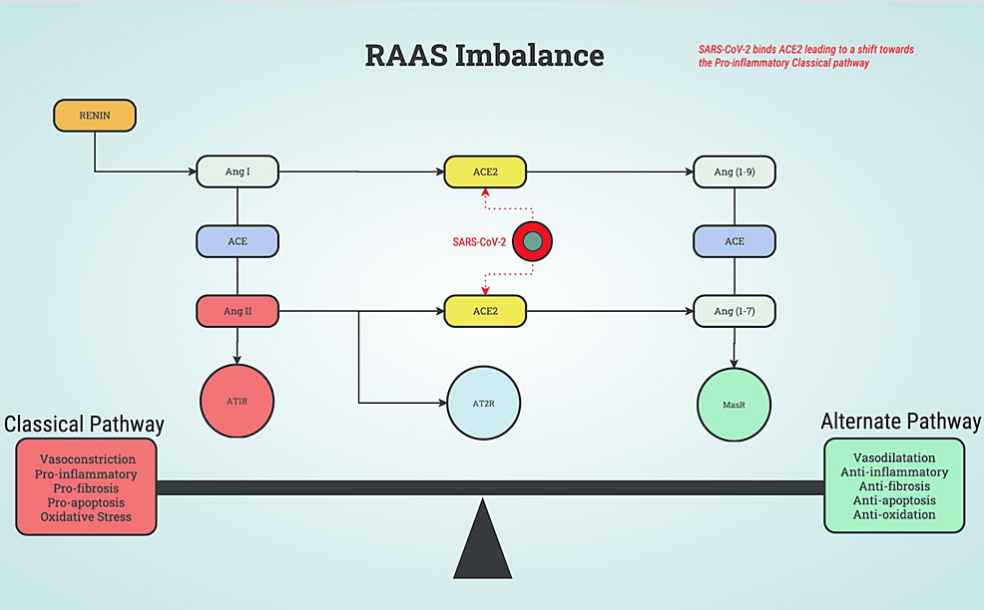
An illustration of SARS-CoV-2 binding to angiotensin-converting enzyme 2 (ACE2). The interaction of SARS-CoV-2 and the ACE2 receptor leads to a shift towards the angiotensin-converting enzyme-angiotensin II-angiotensin II receptor (ACE-Ang II-AT1R) axis classical pathway and promotes vasoconstriction, pro-inflammatory response, pro-fibrosis, pro-apoptosis, and oxidative stress. RAAS: renin-angiotensin-aldosterone system Image credits: Randy Felber

The ACE2 membrane-bound receptor is a key component of the renin-angiotensin-aldosterone system (RAAS) hormone regulation pathway, an essential pathway of blood pressure regulation, in addition to its involvement in inflammation. Two pathways make up the RAAS which are: the classical angiotensin-converting enzyme-angiotensin II-angiotensin II receptor (ACE-Ang II-AT1R) axis and the alternate angiotensin-converting enzyme 2-angiotensin(1-7)-mas receptor (ACE2-Ang(1-7)-MASR) axis. The classical ACE-Ang II-AT1R pathway promotes contraction of blood vessels, electrolyte balance, aldosterone synthesis and release, pro-fibrosis, pro-apoptosis, oxidative stress, and pro-inflammation. On the other hand, the counterregulatory ACE2-Ang(1-7)-MASR pathway promotes vasodilatory, anti-fibrosis, anti-apoptosis, anti-oxidation, and anti-inflammation. In the RAAS, ACE2 is responsible for degrading angiotensin I (Ang I) into Ang-(1-9) and Ang II into Ang-(1-7). As SARS-CoV-2 enters the cell, it causes a downregulation of ACE2, which leads to an imbalance in the RAAS, leaning more towards the pro-inflammatory classical pathway. Figure 1 illustrates the effect of SARS-CoV-2 when it interacts with ACE2. 

The effects mediated by the classical ACE-Ang II-AT1R pathway are largely due to AT1R and its location. AT1R is expressed in the heart, lungs, kidneys, blood vessels, skeletal muscles, brain, liver, skin, and adrenal glands. When AT1R is activated, the downstream effects can lead to cellular dysfunction, such as increased oxidative stress and apoptosis [2]. For example, in the heart, AT1R causes endothelial dysfunction, leading to the promotion of vascular remodeling and the start of atherosclerosis. Cellular hypertrophy arises from the activation of AT1R in cardiomyocytes, whereas the stimulation of AT1R in cardiac fibroblasts promotes extracellular matrix protein synthesis, leading to cardiac fibrosis. Ultimately, the ACE/ACE2 ratio could play a role in patient outcomes, either favoring the cardiotoxic (AT1R) or cardioprotective (MASR) axis of the RAAS system.

Targeting the RAAS system, more specifically the ACE-Ang II-AT1R pathway has been a therapeutic strategy for hypertension, along with preventing heart and kidney damage. Angiotensin-converting enzyme inhibitors (ACEi) and angiotensin receptor blockers (ARBs) are two classes of drugs that inhibit the RAAS and have been debated as possible treatments for COVID-19. Angiotensin receptor blockers prevent ANG ll from binding AT1R, resulting in the inhibition of downstream effects and providing additional benefits to the organ. Despite the potential beneficial effect RAAS inhibitors have, Tetlow et al. discuss how the use of these drugs has been shown to upregulate ACE2 receptors and therefore increase susceptibility to SARS-CoV-2 infection [3]. With growing suspicion of whether or not RAAS inhibitors are beneficial to the COVID-19 patient as well as the development of new variants such as Omicron, many researchers have performed studies to explore the validity of these claims. 

The Omicron variant of SARS-CoV-2 that began to take over in December 2021 has been shown to have increased interactions with the ACE2 receptor via the SARS-CoV-2 spike protein [4]. This could lead to a greater downregulation of ACE2 and the possibility of RAAS inhibitors having a greater role in the treatment of these patients infected with the Omicron variant. In this review, we aim to evaluate the effectiveness of ACEi and/or ARBs as adjunct therapy in patients with COVID-19 due to SARS-CoV-2 infection. This also includes the analysis of possible protective effects RAAS inhibitors could have, as well as their impact on infection rate and disease severity. The results of this review will determine whether the available United States (US)-based studies align with the current national guidelines on whether to continue or discontinue RAAS inhibitors upon infection with SARS-CoV-2.

## Review

Methods

**Figure 2:**
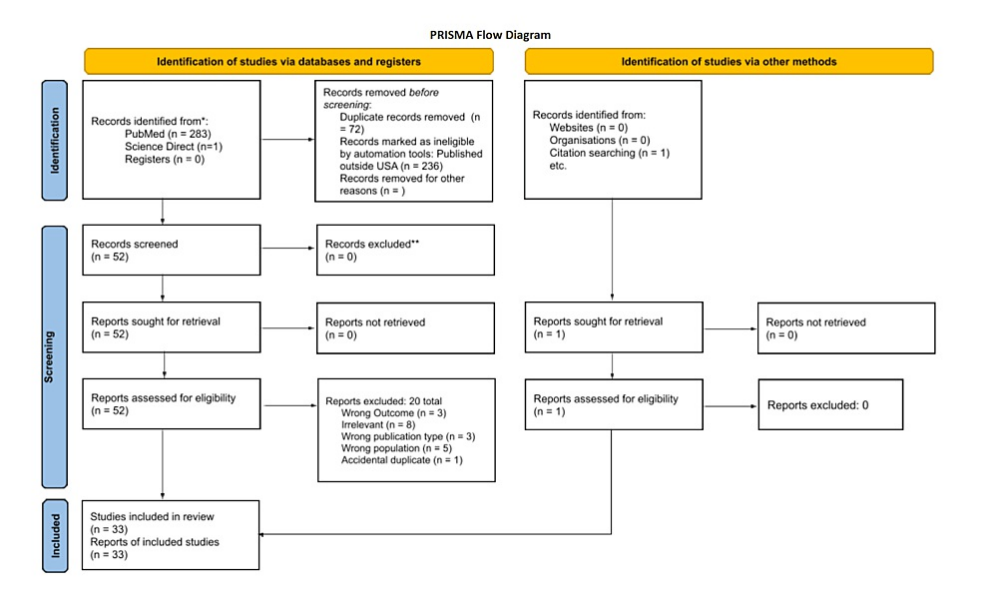
Preferred Reporting Items for Systematic Reviews and Meta-Analyses (PRISMA) diagram to determine studies used to evaluate SARS-CoV-2 binding to angiotensin-converting enzyme 2 (ACE2) with an effect on ACE inhibitor (ACEi) and angiotensin receptor blocker (ARB) utilization.

A PubMed literature search was performed using the following search criteria: “COVID-19 AND cardiovascular disease AND ACEi AND ARB”, “SARS-COVID-19 OR COVID-19, AND ACEi AND ARB AND Infection rate”, “COVID-19 AND ACEi and ARB”, “Omicron BA.1 and BA.2 AND ACE2 OR ARBs”, “Omicron AND ACEi AND ARBs”. This yielded 284 articles, of which 251 were excluded due to being duplicates or published using data from outside the US. This left 33 sources available to use in the final analysis. Figure 2 illustrates a flowchart based on the Preferred Reporting Items for Systematic Reviews and Meta-Analyses (PRISMA) guidelines outlining the study selection process.

Results

Evaluating Clinical Practices According to Current Guidelines

The current guidelines from the American College of Cardiology (ACC), American Heart Association (AHA), European Society of Cardiology (ESC), and Heart Failure Society of America (HFSA) recommend that ACEi/ARB medications should not be initiated or withdrawn from COVID-19 patients with cardiovascular disease. These guidelines also state that each patient should be evaluated individually, and decisions should be made on a case-by-case basis [5]. A meta-analysis and systematic review did not find a significant difference between ACEi/ARB users and non-users with COVID-19 infection in terms of mortality, illness severity, or duration of stay. These findings were in agreement with the ACC/AHA, which recommends continuing the use of ACEi and ARBs in the presence of COVID-19. The study did show a possible protective impact associated with the use of ACEi/ARBs in COVID-19 based on the reduction in C-reactive protein (CRP) and D-dimer in these groups when compared to control groups, but the study suggested that larger sample numbers are required to confirm this effect [6].

An observational analysis found 1,449 patients with a COVID-19 diagnosis using data from a Massachusetts medical group. Results from this study showed that patients under the age of 65 were more likely to experience unfavorable COVID-19 outcomes when they had cardiovascular-related comorbidities. Those on beta blockers, calcium channel blockers (CCBs), and anticoagulants, which they classified as all antihypertensive drugs, did not significantly enhance their risk of severe COVID-19. The study also analyzed ACEi/ARB use in these patients, and their results do not suggest discontinuing ACEi or ARB in the treatment of hypertension to lower the risk of developing severe COVID-19 disease [7].

An observational analysis done in early 2020 evaluated 14,129 patients who had hypertension and then contracted a COVID-19 infection. The use of ACEi or ARBs was not associated with a higher risk of hospitalization or mortality prior to the COVID-19 infection. According to the study's conclusions, patients who have hypertension should continue taking ACEi or ARBs during COVID-19 [8]. To further support the continued use of ACEi/ARB in those hospitalized with COVID-19, a study published in 2022 analyzed 614 hospitalized COVID-19 patients with hypertension and the continued and discontinued use of ACEis or ARBs. In comparison to patients who stopped using ACEi/ARBs, patients who continued the medication had significantly lower rates of intensive care unit (ICU) admission (12% vs. 26%; P = 0.001; odds ratio (OR) = 0.347; 95% confidence interval (CI), 0.187-0.643) and death (6% vs. 28%; P = 0.001; OR = 0.215; 95% CI, 0.101-0.455) [9].

Another study randomly assigned 659 COVID-19 patients with a confirmed diagnosis of mild or severe COVID-19 disease severity at hospital presentation. Patients with moderate disease who remained using ACEis/ARBs had more days alive and outside of the hospital through 30 days than those who stopped taking ACEis/ARBs, in contrast to patients with mild disease. This implies that individuals with intermediate COVID-19 disease severity should continue taking ACEis and/or ARBs [10]. The analysis of data from US Veterans Affairs revealed 9,532 hospitalized patients with COVID-19 infection followed for 60 days. An increased risk of death was linked to stopping ACEis (OR = 1.4; 95% CI, 1.2-1.7). A lower risk of death was linked to starting (OR = 0.3; 95% CI, 0.2-0.5) or maintaining (OR = 0.6; 95% CI, 0.5-0.7) ACEis. The safety of continued ACEi and ARB medication in patients with COVID-19, where needed, is supported by recent randomized clinical trials. This study expands on these conclusions by suggesting that metformin, ACEi, and ARB therapy may be continued or started to improve COVID-19 survival [11].

Clinical Outcomes of COVID-19 With the Use of ACEi/ARB

A retrospective cohort study looking at positive COVID-19 reverse transcription polymerase chain reaction (RT-PCR) patients from March 1 to April 17, 2020, found that advanced age along with an increasing number of comorbidities are independent predictors of in-hospital mortality for patients positive for COVID-19 in the hospital. Another finding showed that nonsteroidal anti-inflammatory drugs (NSAIDs) and ACEi/ARB use prior to admission are not associated with an increase in mortality or renal failure [12].

Over 2,900 COVID-19 patients who were hospitalized in six hospitals in southern California, US, between March 2020 and August 2021 were included in a multicenter retrospective observational study. The main finding concerned the impact of pre-hospital ACEi and ARB use on COVID-19 patients' in-hospital mortality. The usage of ACEi (hazard ratio (HR) = 1.226; 95% CI, 0.989-1.520) or ARB (HR = 0.923; 95% CI, 0.701-1.216) was not independently linked with increased mortality once confounding variables were taken into account in the multivariate analysis [13].

Hospitalized African American patients positive for COVID-19 who either died in the hospital or survived to discharge between March 2 and May 22, 2020, were divided into two groups: those on ACEi/ARB at baseline and those not on them. After analysis, it was shown that the baseline use of ACEi/ARB does not worsen outcomes in hospitalized COVID-19 African American patients [14].

Further analysis of over 20 studies that compared patients with COVID-19 to a control group of COVID-19 patients who did not take ACEis or ARBs revealed that concurrent ACEi/ARB use is not associated with COVID-19 disease severity or patient mortality, even with a pooled analysis of both unadjusted data and adjusted data (studies with matched controls) and taking into account factors such as study bias risk via meta-regression and sensitivity analyses [15].

Taking a different look at the US population, investigators discovered that using an ACEi/ARB in patients with COVID-19 was not linked to higher mortality or other negative outcomes within a national cohort of US veterans. However, further research should look at the underlying mechanisms and a possible link between the use of ACEIs and sepsis, which could lead to increased susceptibility to negative COVID-19 outcomes [16].

The possibility of using renin-angiotensin-aldosterone system inhibitors (RAASi) as a protective therapy that could positively impact the outcome of those positive for COVID-19 has been extensively examined in the medical field. Sattar et al. reviewed a total of 49 observational studies comprising 83,269 individuals with COVID-19 and divided them into groups based on RAASi use (RAASi n = 34,691; non-RAASi n = 48,578). The result of this review shows that continued RAASi use may lead to good COVID-19 results, including seropositivity and viral elimination. Although they are not linked to increased mortality or symptoms getting worse, COVID-19 is not a contraindication to continuing with or stopping RAASi [17].

Stanford Hospital evaluated 1,023 COVID-19 patients who had been diagnosed by RT-PCR as of April 8, 2020. The use of ACEi or ARB was not linked to a higher risk of hospitalization, admission to an ICU, or death. Those using ACEi or ARB had a decreased likelihood of hospitalization compared to people with previously documented medical histories. Patients with hypertension taking an ARB had a decreased risk of hospitalization compared to those not taking an ACEi or ARB (OR = 0.09; 95% CI 0.01-0.88; P = 0.0381) [18].

Between March 25 and June 22, 2020, there were 841 patients with COVID-19 at the University of Chicago Medical Center. Both ACEi/ARB and CCB exposure were not related to any effect on mortality after controlling for demographic factors and confounders, whereas ACEI/ARB exposure was associated with a 42% reduction in the risk of ICU admissions. Using ACEi/ARB and CCB together was linked to a statistically significant (45%) decrease in ICU admissions. There were 453 patients with a personal history of hypertension; out of these, 85 (18.76%) were taking an ACEI or an ARB. After analyzing these patients, the use of ACEis was linked to a 71% decrease in in-house mortality [19].

Restricting studies to hypertensive patients only, the analysis revealed that the use of ACEi and/or ARB was associated with a significant decrease in in-hospital mortality when compared to not using ACEi or ARB. The analysis also revealed that the use of ACEi and/or ARB did not affect in-hospital mortality in non-hypertensive patients. The protective effect of ACEi/ARB on patients with COVID-19 has been reported, including reduced severity of COVID-19 pneumonia by preserving hypoxic vasoconstriction, limited deterioration of renal function, and protection against myocardial injury. These results support continued use of the medications regardless of a prior history of hypertension [20].

A large study of 178 US hospitals evaluated 2,726 hospitalized patients with confirmed COVID-19 between January 1 and April 1, 2020. Before hospitalization, 352 (12.9%) patients were taking an ARB, while 398 (14.6%) patients were taking an ACEi. Angiotensin-converting enzyme inhibitors before admission were independently linked to a lower requirement for mechanical ventilation and mortality. When compared to the non-renin-angiotensin-aldosterone system blockage (RAASb) group, patients who took ARBs had a lower likelihood of needing mechanical ventilation (7.4% vs. 12.2%). When compared to the non-RAASb group, ARB use before admission was also independently related to a lower need for mechanical ventilation and death [21].

In 197 hospitals across the US, symptomatic emergency department patients were collected; renal failure occurred in 548/13,813 (4.0%) and respiratory failure in 2,485/13,880 (17.9%) patients. In SARS-CoV-2-positive patients, baseline ACEi/ARB was linked to a 25% reduced mortality rate after controlling for these covariates [22].

In early 2020, 6,218 patients with COVID-19 were admitted to Mount Sinai hospitals in New York City. The investigators examined whether ACEi/ARB use in outpatient settings and hospitals was related to COVID-19 in-hospital mortality in a cohort of African Americans as opposed to non-African Americans. The in-hospital use of ARBs was linked to a significant drop in in-hospital mortality among African American patients who tested positive for COVID-19 [23]. After analyzing these available research studies, it is clear that the use of ACEi/ARB can provide a limited but positive impact on COVID-19 outcomes. Further research is needed to examine the long-term effects of COVID-19 on the human body and whether ACEi/ARB can provide a lasting protective effect.

Contrary to most research, a few studies were found to show evidence that ACEi/ARB use in COVID-19 patients worsened the outcome. Patients positive for SARS-CoV-2 with diabetes mellitus and hypertension who are commonly prescribed ACEi/ARB appear to have worse outcomes more frequently, which may be related to ACE2 receptor upregulation in airway alveolar epithelial cells. The study concluded that there is currently no convincing evidence to indicate a relationship, but given the pandemic's rapid evolution, their findings could change [24].

A cohort of individuals with hypertension taking ACEis, ARBs, or other hypertensive medications consisted of 1,059,474 total episodes of acute viral respiratory infections, with 653,797 involved with ACEi/ARBs and 405,677 with other hypertensive medications. A larger relative risk of mortality was found in hypertension patients taking ACEis or ARBs compared to hypertensive patients taking other hypertensive medications in a 2019/2020 and 2017/2018 comparison analysis. In addition, poorer outcomes were increased in hypertensive patients taking ACEis or ARBs compared to those taking other hypertensive medications during the COVID-19 pandemic [25].

Aside from the effect ACEis/ARBs have on COVID-19 outcomes, there were a few studies that looked at the possibility of these medications decreasing the chance of being infected with SARS-CoV-2. A meta-analysis performed in 2020 displayed that the use of ACEi/ARBs was associated with a statistically non-significant decreased risk of getting serious disease compared to non-users in a pooled analysis of four of the 16 trials. There was a statistically non-significant connection between ACEi/ARB use and lower risks of mortality as compared to non-users in a pooled analysis of six trials [26].

Taking another look at US veterans, over 436,000 veterans tested for COVID-19 infection were evaluated. Patients with mild cognitive impairment (MCI) or Alzheimer’s disease (AD) with or without cognitive impairment and the impact of ACEI/ARB medication were examined. Patients with AD/MCI who were on angiotensin II receptor blockers had a significantly decreased probability of developing COVID-19. An interesting result of this study showed that there is a significant association between AD and an increased risk of COVID-19 infection and mortality [27]. A further review of this association specifically could yield some interesting and clinically significant results that may impact the way patients with AD are treated. 

Iwanski et al. performed a stem-cell-based experiment to test the speculated hypothesis that those patients taking ACEi/ARB have increased susceptibility to SARS-CoV-2 due to the ability of the virus to enter cells via the ACE2 receptor. Human pluripotent stem cell-derived cardiomyocytes (hPSC-CMs) and human endothelial cells (hECs) exposed to SARS-CoV-2 revealed remarkable differences in immunity genes, response to infection, and cellular structure. The susceptibility of hPSC-CMs and hECs to SARS-CoV-2 infection was unaffected by pre-treatment with losartan or lisinopril, two commonly prescribed antihypertensive drugs. When combined with recently published multicenter trials, these findings show the toxic effects of SARS-CoV-2 in hPSC-CMs/hECs and imply that antihypertensive medication therapy alone does not affect SARS-CoV-2 infection [28].

Renin-Angiotensin-Aldosterone System Blockade and COVID-19: Pro-inflammatory and Anti-inflammatory Balance

To quantify the influence of chronic diseases or aging, SARS-CoV-2 infection, and RAAS therapy on the balance between the inflammatory (AT1) and anti-inflammatory (Mas) arms of the RAAS, a simulation model was performed to do just that. First, the simulation showed that the ratio of Mas to AT1 receptor occupancy is greatly increased by ACEi/ARB therapy prior to SARS-CoV-2 infection. As a result, the anti-inflammatory arm of the RAAS would already be significantly more dominant in individuals who were already taking an ACEi/ARB prior to infection. Second, changes in ACE2 expression with comorbidities such as diabetes, hypertension, or aging have relatively minor effects on the Mas-AT1 receptor ratio. Third, it is hypothesized that pro-inflammatory changes in the Mas-AT1 ratio brought on by SARS-CoV-2 infection will be less pronounced than anti-inflammatory changes brought on by ACEi/ARB. Finally, predicted changes in the Ang(1-7) production rate with ACEi/ARB therapy, comorbidities, or infection were all minimal compared to the exogenous Ang(1-7) infusion rates demonstrated experimentally to protect against acute lung injury, suggesting that changes in the ACE2-Ang(1-7)-Mas arm may not be significant enough to play a role in COVID-19 pathology [29]. 

To determine if the Omicron variant is any different, a molecular dynamics simulation study investigated the subvariants BA.1 and BA.2 of the Omicron variant of SARS-COV-19 and their increased interactions with the peptidase domain (PD) of the ACE2 receptor via the receptor-binding domain (RBD) of the SARS-CoV-2 spike glycoprotein. These variants exhibit a more dispersed interaction network and make an increased number of salt bridges and hydrophobic interactions with PD compared to the wild-type RBD, providing molecular biochemical reasoning for Omicron’s increased affinity to ACE2 receptors [4]. With this finding, it is speculated that due to the increased affinity to the ACE2 receptor, Omicron will have an increased infectivity, especially in those taking ACEi/ARBs, which can theoretically increase ACE2 receptors on the surface of cells.

Limited studies are looking at the Omicron variant specifically, but a retrospective study looking at in-hospital mortality of patients with COVID-19 during the Omicron period (January to May 2022) analyzed risk factors and markers in 74 COVID-19-positive patients. The study found that high comorbidity burden, high levels of neutrophil-to-lymphocyte ratio, and undertreatment with ACEI/ARBs were the main prognostic indicators of in-hospital mortality. Using this information, testing for variant-specific cases can enhance risk stratification in hospitalized patients and reduce mortality in high-risk patients [30].

## Conclusions

Using its membrane-bound spike protein, SARS-CoV-2 is known to bind, fuse, and enter specific cells via the ACE2 receptor, resulting in the current COVID-19 pandemic. These receptors are found on a variety of cells within the cardiovascular, respiratory, nervous, and renal systems. Due to the widespread use of ACEis and ARBs and the pervasiveness of the COVID-19 pandemic, the clinical association between patients who utilize these medications and experience infection by SARS-CoV-2 is explored. According to the majority of available clinical research studies, ACEis and ARBs should not be discontinued or initiated in cardiovascular disease patients infected with SARS-CoV-2. The concurrent use of ACEis and ARBs with COVID-19 treatments was not associated with increased patient mortality or disease severity and showed limited protective effects. Patients on ACEis or ARBs were found to have a decreased risk of hospitalization, reduced severity of COVID-19 pneumonia, a lesser need for mechanical ventilation, and an overall reduction in mortality rate. The majority of current clinical research supports the guidelines reported by the ACC, AHA, ESC, and HFSA which state that ACEi and ARB medications should not be withdrawn from or initiated in patients with cardiovascular disease who are infected with SARS-CoV-2. Looking forward, COVID-19 variants and their fruition need constant surveillance, as alterations such as the increased affinity between ACE2 and the viral spike protein found in some Omicron variants may cause increased infectivity. More research needs to be conducted on the exact interplay between COVID-19 and ACEis/ARBs to give providers in the community confidence when treating patients within this subgroup of the population.
